# A New Suggestion about Nerve Centres

**Published:** 1889-01-12

**Authors:** 


### WITHIN THE WARDS.

### A NEW SUGGESTION ABOUT NERVE CENTRES.

It has long been considered by physiologists that the brain is an organ which contains many different and more or less functionally independent centres of energy. The energy is distributed by the various nerves to the different organs and parts of the body, where it is required for special purposes. For example, the eye sees by means of a spread-out nerve ending. The origin of the nerve is in the brain, and that part of the brain from which the nerve starts is the sight centre. Similarly the lower jaw is worked by an arrangement of muscles. The muscles are supplied with energy by motor nerves, and the motor nerves, which terminate in the muscles, start from the brain. That portion of the brain from which they originate is the nerve centre for the muscles of the lower jaw. If the brain really does consist principally of nerve centres, and if each of these be a centre, all of whose cells have but one function, and that the function proper to the particular centre, then it follows that the complete destruction of any given centre would entirely and for ever abolish the function of that centre. In other words, according to some physiologists, the whole brain is really an aggregate of several little brains, each of them doing its own work, and quite incapable of taking any part in the proper work of any other little brain. The eye centre contains no ear cells, and cannot therefore help the ear centre at a pinch; the ear centre contains no taste cells, and cannot on an emergency help the centre for taste, or partially take its place. These, we say, are the generally received views; and there is good reason for believing that they express substantial truth. But do they express *all* the truth about the brain and its several nerve centres? Dr. Brown-Sequard, who is one of the most famous as well as one of the oldest of experimental physiologists, has recently come forward with a fresh suggestion. Each nerve function, of taste, or smell, or hearing, or movement, is carried out by means of energy, generated by particular nerve cells whose main or exclusive business it is to generate that particular kind of nerve force and no other. So far his view is in accordance with that generally received. But, whereas it has previously been held that these particular cells are grouped together in masses consisting almost exclusively of like cells, so as to form a definite nerve centre, having one definite function and no other, Dr. Brown-Sequard thinks, for reasons which he gives, that the cells which perform like functions, are not exclusively grouped together in distinct masses, but distributed generally throughout the brain. The idea, though probably not entirely new, is newly stated and re-argued from the present standpoint of knowledge. Perhaps, however, the true view will be found to lie between the old and the new. Probably most of the nerve cells which have a certain function are grouped together in more or less exclusive masses; but it may be that, in order to provide for the due performance of function in case of the destruction of one or more of these centres, there are scattered throughout the brain-mass single cells, or very small groups of cells, of the same kind and performing the same function. Thus, we should have a kind of collateral performance of brain-function in case of need, exactly as we have a collateral blood circulation coming in to perform the function of a destroyed artery. The subject is one of far-reaching importance as bearing upon prognosis and treatment. Dr. Brown-Sequard's view will explain many difficult problems, both of the laboratory and of the bed-side.

